# From Paths to Routes: A Method for Path Classification

**DOI:** 10.3389/fnbeh.2020.610560

**Published:** 2021-01-21

**Authors:** Andrea Gonsek, Manon Jeschke, Silvia Rönnau, Olivier J. N. Bertrand

**Affiliations:** Neurobiology, Bielefeld University, Bielefeld, Germany

**Keywords:** bumblebee, clustering, route, classification, clutter, navigation

## Abstract

Many animals establish, learn and optimize routes between locations to commute efficiently. One step in understanding route following is defining measures of similarities between the paths taken by the animals. Paths have commonly been compared by using several descriptors (e.g., the speed, distance traveled, or the amount of meandering) or were visually classified into categories by the experimenters. However, similar quantities obtained from such descriptors do not guarantee similar paths, and qualitative classification by experimenters is prone to observer biases. Here we propose a novel method to classify paths based on their similarity with different distance functions and clustering algorithms based on the trajectories of bumblebees flying through a cluttered environment. We established a method based on two distance functions (Dynamic Time Warping and Fréchet Distance). For all combinations of trajectories, the distance was calculated with each measure. Based on these distance values, we grouped similar trajectories by applying the Monte Carlo Reference-Based Consensus Clustering algorithm. Our procedure provides new options for trajectory analysis based on path similarities in a variety of experimental paradigms.

## 1. Introduction

Finding a location in an unknown environment can be a daunting time- and energy-demanding task. In contrast, returning to a known location is much easier than finding it for the first time. To return to an already known location, animals and artificial agents alike can move along habitual routes. Forming and following of routes has been observed in numerous taxa; from insects (Lihoreau et al., 2011; Woodgate et al., 2016; Buatois and Lihoreau, 2016; Woodgate et al., 2017) to mammals (Hurlebaus et al., 2008; Pfeiffer and Foster, 2013); thus, it is a wide-spread strategy to navigate in a familiar environment. Despite the large number of taxa following routes, it remains little understood how routes are established and followed.

Thanks to the rise of miniature embedded tracking devices (Nagy et al., 2010; Genzel et al., 2018; Greif and Yovel, 2019), and high-throughput computational methods, tracks of individual animals in various natural habitats (Graving et al., 2019) have become more wide spread in recent years. With this expanding collection of paths gathered by scientists, there is a growing need for efficient data-analysis pipelines to identify, classify, and compare different paths across taxa, species, or individuals.

There is a distinction to be made between an animal's path and a route. A path specifically describes the animal's trajectory of movement, while the route can be visualized as a string around which different paths meander. Depending of the consistency of the paths taken among different runs, a potential route may not easily be recognizable to an observer. However, when many paths are observed and clearly show a common overarching structure, one may conclude that the animals are following the same route.

To date, different paths were visually grouped into different routes. However, this may lead to unintentional biases toward a preferred hypothesis. Alternatively to a qualitative assessment, one may cluster paths numerically. Paths belonging to the same route would share similar descriptions, be they their average speed, their sinuosity, or spatial similarity among paths. Therefore, we aim at finding descriptions of paths to group them into common routes. During the last century, numerous methods comparing two paths have been developed and refined (see for review Magdy et al., 2015), yielding similarity measures between paths. Therefore, on the one hand we will try to cluster paths based on their characteristics (such as average speed, or positional spread); on the other we will try to cluster paths based on paths similarity measures. With both descriptions of paths (flight characteristics and path similarities) we attempt to identify clusters in the data.

Numerous techniques have been developed to identify clusters in data. Many clustering techniques require to choose the number of clusters beforehand. Others address this problem by using metrics to determine an appropriate number of cluster [e.g., Monti consensus clustering, (Senbabaoğlu et al., 2014), Non-negative Matrix Factorization (Lee and Seung, 2001) or k-means with Ward cost function (Braun et al., 2010)]. Such algorithm may however bias the results toward higher or lower number of clusters. A novel method, named Monte Carlo reference-based consensus clustering (M3C), allows to cluster the data and determine the number of clusters from the data while avoiding a bias toward a higher number of clusters (John et al., 2020). This is performed by statistically testing a given number of clusters against the null hypothesis of having only one cluster.

We propose to combine a clustering algorithm (here M3C) and a number of features describing paths, be it flight characteristics (e.g., average speed) or similarity measures, to identify potential routes followed by animals. To illustrate this combination, we use behavioral data of bumblebees, *Bombus terrestris*, known for their route following skills (Lihoreau et al., 2011), flying through a heavily cluttered environment. We compare the trajectories of bees through an obstacle parkour by using two similarity measures [Dynamic Time Warping (Salvador and Chan, 2004) and Fréchet distance (Fréchet, 1906; Magdy et al., 2015)], derive the number of potential routes and associate the individual trajectories to their corresponding route by using the M3C clustering algorithm. Furthermore, we classify trajectories based on flight characteristics, such as the average speed, to assess whether several characteristics are sufficient descriptors to identify routes from paths. The clustering algorithm may yield ambiguous results. We complemented the clustering outcomes with a method to visualize high dimensional data. Such visualization allow to disambiguate between different clustering outcomes. Finally, we discuss the potential use of alternative similarity measures and how to place novel trajectories into an existing classification.

## 2. Materials and Methods

### 2.1. Data Acquisition

#### 2.1.1. Animal and Hive

We used two healthy hives of *Bombus terrestris* provided by Koppert B.V., The Netherlands. Bumblebees were transferred into a 30 × 30 × 30 cm^3^ acrylic box. Inside the hive box, bumblebees were provided with pollen. Before starting the experiment, the bumblebees got 1 week of habituation time to access the foraging chamber at any time. In the foraging chamber, bumblebees were provided with feeders containing sucrose solution (0.5kg/L). After habituation, we could usually observe bumblebees flying in a direct manner between foraging chamber and hive. These bees, likely to be foragers, were marked to track their individual learning progress. To this end, the animals were captured and restrained on their way back to the hive. A small colored plastic tag was fixed with resin on the animals' thorax. After the marking procedure, the bumblebee was placed close to the hive entrance.

#### 2.1.2. Procedure

**Figure 1:**
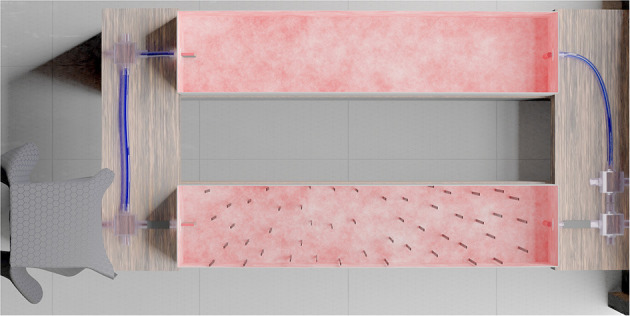
Experimental setup shown from above. The hives were kept in an acrylic box shown on the left. Bees were allowed to forage on sucrose solution in the foraging chamber (to the right, not shown), which could be reached by traveling through a tube and tunnel (top) system. Marked foragers could, upon exiting the foraging chamber, be re-directed into the experimental tunnel (bottom), where 49 vertical objects form a complex cluttered environment, which they had to cross to return to their hive.

The habituated bees were allowed to travel through a foraging tunnel (140 × 30 × 30 cm^3^) connected to the hive box and a foraging chamber via 2.5 cm diameter tubes and acrylic boxes (see Figure 1). The walls of the tunnel were covered with a red and white 1/*f* noise pattern (as in (Ravi et al., 2019)). When an individually marked bumblebee returned from the foraging chamber, it was rerouted by using small acrylic gates into an experimental tunnel, parallel to the foraging tunnel. Only one bee at a time was permitted to cross the experimental tunnel.

The experimental tunnel, used for individual training and recording, contained 49 vertical objects (29.5 × 1 cm^2^) suspended from the ceiling and creating a cluttered environment. The objects were made of red acrylic that blocks light below a wavelength of 650 nm. Objects were placed as in Figure 1. Five cameras (Basler acA2040-90umNIR) with red filters (Heliopan RG715) viewed the tunnel from different perspectives, and allowed recording the bee's behavior.

A recording started as soon as the bee crossed the infrared-light barriers placed before to the tunnel entrance, and stopped as soon as the bee crossed the light barriers after the tunnel exit. While recording, the tunnel was illuminated from below by light filtered through 650 nm cutoff low-pass acrylic, so that the objects were transparent for the cameras but were perceived as dark by the bumblebees (Dyer et al., 2008).

#### 2.1.3. Trajectories

Inbound flights of individually marked bumblebees were recorded while they were flying through the clutter. The calibrated cameras recorded at 60 frames per second. Each bumblebee was recorded ten times. The frame-wise position of the recorded bee was triangulated using flydra (Straw et al., 2011).

**Figure 2:**
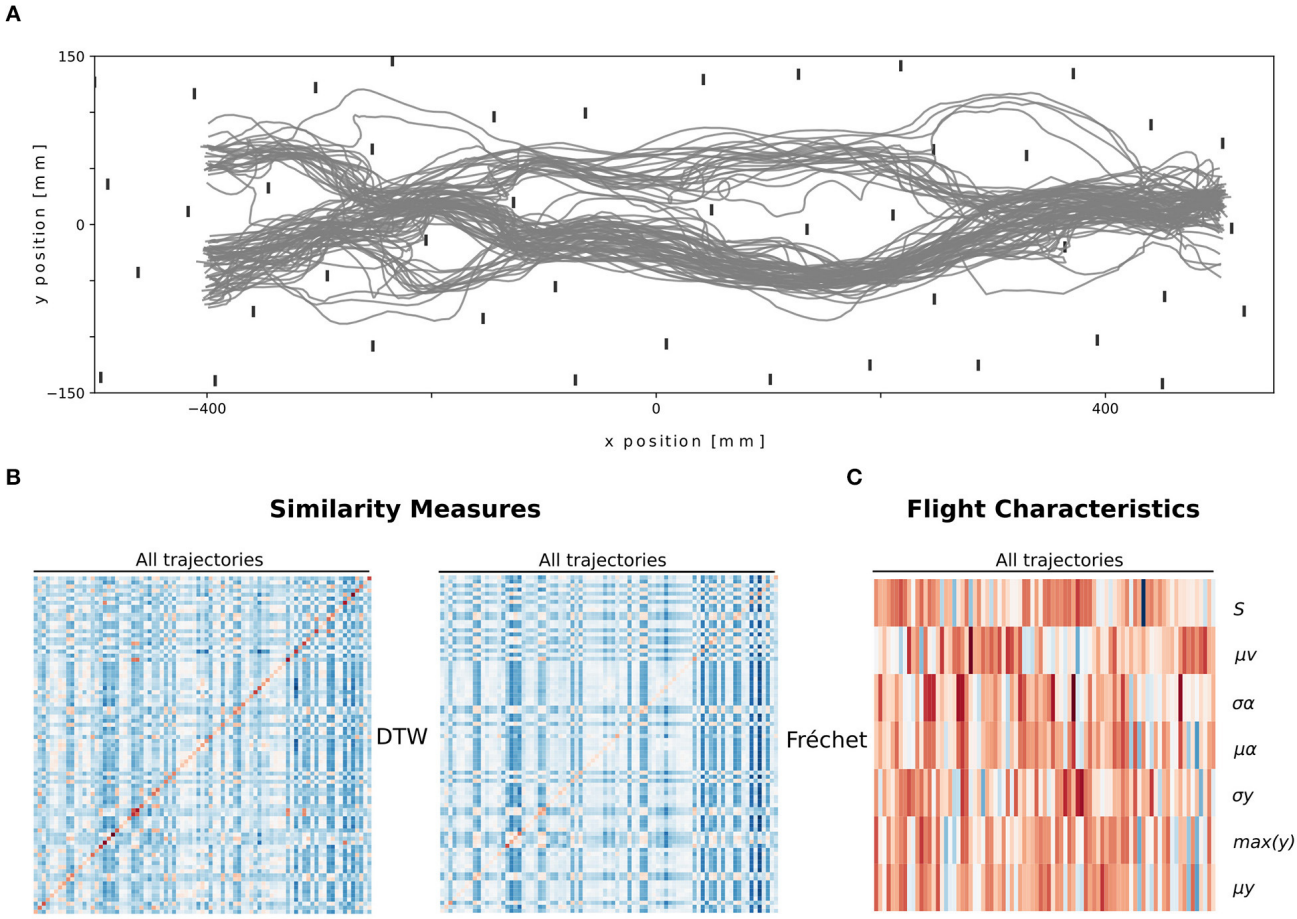
Overview of the trajectories **(A)** and their descriptions, path similarity **(B)** and flight characteristics **(C)**. **(A)** Top view of all unclassified trajectories that were used. **(B)** Heatmap of normalized distance values of both similarity measures (DTW and Fréchet), where the columns represent the trajectories, and the rows the respective paired trajectories for both measures. **(C)** Heatmap of normalized flight characteristic values. The columns represent the trajectories and the rows depict the flight characteristic values.

Afterwards, the trajectories were manually reviewed to check for possible errors. Only trajectories after the fifth trial were considered. In addition, since the setup was invariant along the altitude (i.e., the z-dimension), we reduced the trajectories to their planar projection. We selected trajectories during which bees entered, swiftly crossed, and exited the tunnel. We used a total of 83 trajectories from 27 different individuals (see Figure 2).

### 2.2. Path Clustering

Our aim was to group trajectories into distinct routes. The trajectories were not directly grouped to each other based on the time course of their x,y coordinates, but reduced to a certain number of features, be it flight characteristics (e.g., average speed) or similarity measures (see section 2.2.2 below). This grouping is akin to the problem of identifying clusters, where each cluster of trajectories would correspond to a route.

#### 2.2.1. Flight Characteristics

Along a given route, the bee may fly slower than along another route, because for example obstacles might be closer to the bee Baird et al. (2005). The bee may also decide to follow one wall of the tunnel or to center in it (Serres et al., 2008). Thus the maximal, average, and standard deviation of lateral position may be good predictors of a route. Finally the average and standard deviation of the gaze direction, as well as the traveled distance divided by the shortest distance between the start and the end of the bee's path (i.e., the sinuosity), inform about the overall flight direction and how much the bee meandered in the clutter.

Seven flight characteristics were used to describe each bumblebee's flight trajectory: the average speed μ_*s*_, the lateral position μ_*y*_ of the average trajectory, gaze direction μ_α_ in the tunnel, the standard deviation of the lateral position σ_*y*_ and of the gaze direction σ_α_, the maximal lateral position max(*y*), and the sinuosity.

#### 2.2.2. Path Similarity

Our second method to describe each path was based on similarity measures of their structure. Several functions can evaluate the similarity between two trajectories (Magdy et al., 2015; Su et al., 2020). These functions yield a distance which is the inverse of the similarity between the two trajectories.

Since animals may meander differently along a route, the selected similarity measures between paths must take into account divergent path lengths and keep the traversed locations ordered along time. We considered two measures: a variant of Dynamic Time Warping (DTW) and the Fréchet distance. DTW minimizes the sum of absolute differences between two trajectories, whereas Fréchet identifies the shortest distance between two trajectories that is sufficient to connect points along the trajectories. DTW and Fréchet thus capture different similarities between trajectories, and can be regarded as a global and local measures, respectively (see also Supplementary Figure 1).

The two distance functions required numerous computations, because they iterated through individual observations for each trajectory pair. To reduce the computational cost for the similarity measures, we re-sampled the trajectories as follows: The trajectories were interpolated and afterwards down-sampled to achieve equal distances between neighboring points, in order to keep the shape of the trajectory. The distance between the points was the median speed across all trajectories.

##### 2.2.2.1. Dynamic Time Warping and FastDTW

Dynamic Time Warping (DTW) was one of the similarity measures between two temporal sequences, here two trajectories (Salvador and Chan, 2004). To illustrate this measure, we may picture two strings with knots laid flat on a table. Our goal is then to connect the knots from one of the strings to the other one using the minimum amount of connecting materials. The connections are not allowed to cross each other, we try to make them as short as possible, and the first knots on the each of the strings are connected to each other. DTW is an algorithm that finds such connections between the strings. In our case, the knots are the observed bee's positions, and the strings are the time axes of the trajectories (see also Supplementary Figure 1). Therefore, DTW captured similarities by working on the full paths (i.e., global measure of path similarity).

The computational demands for this function scaled quadratically with the length of the trajectories and was therefore inefficient to use with long trajectories. FastDTW linearly approximates DTW by using a multi-level approach that recursively projects a solution from a reduced resolution and then refines the projected solution (Salvador and Chan, 2007).

##### 2.2.2.2. Fréchet Distance

The Fréchet distance is a spatial similarity measure that can be best described intuitively as a person walking a dog (Fréchet, 1906). They are connected by a retractable leash and are walking on different paths. Assuming that both the person and the dog are allowed to travel with different speeds, but are not allowed to backtrack their path, the Fréchet distance describes the minimal length the leash would need to have to connect both throughout their journey (see also Supplementary Figure 1). Therefore, Fréchet captured similarities with an extremum function (i.e., a local measure of path similarity). It took into account the location of points, as well as their order, but did not shift points along their time axis.

#### 2.2.3. Monte Carlo Reference-Based Consensus Clustering Algorithm

We clustered the path descriptions (either flight characteristics or path similarity) by using the Monte Carlo Reference-based Consensus Clustering algorithm (henceforth called “M3C”). M3C solves a common problem of selecting a suitable number of clusters and also introduces formal hypothesis testing, by generating random data to get an estimate of a random Gaussian distribution.

M3C runs the clustering algorithm multiple times, for each number of cluster *K*, resulting in potentially different partitioning of the data. A consensus is created based on the different runs (Vega-Pons and Ruiz-Shulcloper, 2011). M3C builds a consensus matrix showing the probability of two samples being part of the same clusters. A very high and a very low probability indicate a small ambiguity whether the cluster allocation is correct. The consensus matrix is used to create the cumulative distribution function (CDF) curve. An ideal CDF curve has a flat shape, because ideally only very small and very high probabilities are noted in the consensus matrix. A proportion of ambiguous clustering (PAC) can be derived from the CDF curve. The PAC score quantified the ambiguity of cluster assignments between clustering runs based on the cumulative distribution function (CDF) of the consensus matrix (see Supplementary Figures 3A,B, Figures 1B,C in John et al., 2020).

The lower left portion of the CDF curve represents sample pairs that are rarely clustered together, and the upper right part represents those that are almost always clustered together, whereas the middle segment represents sample pairs with ambiguous assignments in different clustering runs. The PAC-score quantified the middle segment of the CDF curve. It was defined as the fraction of sample pairs with consensus indices falling in an interval between *U*_1_ and *U*_2_, where *U*_1_ is a value close to 0, and *U*_2_ a value close to 1 (usually 0.1 and 0.9). Thus, a low PAC-score and therefore a flat middle segment indicated a low rate of discordant assignments across clustering runs.

Furthermore, M3C assessed whether the PAC score for a given number of *K* is significantly lower than that for a single cluster *K* = 1. M3C simulated data sets to get null distributions of PAC scores for K = 1 and tested the following hypothesis.

H0: the PAC score does come from a single Gaussian cluster

The alternative hypothesis was:

HA: the PAC score does not come from a single Gaussian cluster

This hypothesis testing was done for each *K* (here ranging from 2 to 10 routes) and thus provided a *p*-value for each *K*. When a PAC score was at a low local minimum and its associated *p*-value is below 0.05, the path descriptions significantly clustered, indicating distinct group of paths (i.e., routes).

The procedure to decide on a suitable number of clusters was not unambiguous. Indeed more than one *K* may have a low PAC score associated with a *p*-value below 0.05. To disambiguate between two *K* we visualized the cluster by projecting the data (a high dimensional space) using t-distributed stochastic neighbor embedding (t-SNE) on a 2D space. After projection, clusters become visible and may allow to visually disambiguate between clustering outcomes.

#### 2.2.4. Comparison of Path Clustering

Our method used a free parameter: the re-sampling coefficient. We investigated the effect of the free parameter for a range of speeds *s* ∈ [2, 11] mm/frame. The choice of the re-sampling coefficient may change the clusters of trajectories. Therefore, we reran our clustering algorithm with different coefficients. We, then, compared the clustering results from the re-sampling coefficient *s* ≠ 6 mm/frame to the one with *s* = 6 mm/frame.

The clustering results were compared by building a confusion matrix with the reference being *s* = 6 mm/frame as follows. A given pair of trajectories (A and B) belonged to the same cluster when trajectories were re-sampled with *s* = 6 mm/frame and also when the trajectories were re-sampled with *s* ≠ 6 mm/frame. Hence, we had a true positive. Similarly, two trajectories (A and C) did not belong to the same cluster with both re-sampling coefficients. Thus, we had a true negative. Additionally, when two trajectories (A and D) belonged to the same cluster with the reference re-sampling (resp. the tested re-sampling) coefficient but did not with the tested re-sampling (resp. reference re-sampling) coefficient, we had a false negative (resp. false positive).

We used a precision score from the confusion matrix derived from whether pairs of trajectories clustered or did not cluster together. The precision score was the amount of true positives divided by the sum of the true positives and false positives.

The resulting precision score may be due to chance. Therefore, to interpret the precision scores statistically, we simulated 100 random clustering results. We randomly assigned trajectories to a given cluster (from two to ten clusters). We calculated the precision score for the 100 random clustering *T* and derived from their distribution the probability that our observed precision score (or a higher score) *t* came from this distribution *p* = *P*(*T* ≥ *t*|*H*). In this case, the distribution served as null hypothesis, where the critical value for α = 0.05 can be inferred from the precision score value at the 95*th* percentile of the distribution. Consequently, the *p*-value for our observed precision score was determined by the cumulative probability of all values beyond that point, i.e., the area under the graph between the 95*th* and 100*th* percentile of the distribution.

## 3. Results

We proposed a method to identify routes based on quantitative descriptions of individual trajectories. To illustrate our method, we used paths from bumblebees flying in a cluttered environment. Our procedure consisted of four steps:

1. Describing the trajectory: path similarity or flight characteristics
2. Deriving the number of routes
3. Validation of route number
4. Visualization of the routes.

### 3.1. Describing the Trajectories of Bumblebees

We described the bumblebees' paths (Figure 2A) by first using flight characteristics. Each trajectory is thus described by seven values. We observed that some trajectories share multiple characteristics and thus may form clusters of paths (Figure 2C).

Second, we described the bumblebees' paths by using path similarities. We used two measures Fréchet distance and DTW on 83 trajectories. Each trajectory is thus described by 83 values for each measure. The path distances contain two diagonals with zeros. These values correspond to the similarity of each trajectory with itself. Blocks of similar values are present, thus potentially different clusters (Figure 2B).

These two descriptions will be independently fed to the M3C algorithm.

### 3.2. Determining a Significant Number of Routes

When looking at bees' trajectories in the clutter, it seems that paths visually cluster along specific “routes” (Figure 2). Using our descriptions of trajectories (flight characteristics or path similarities) we applied the M3C algorithm to identify groups of trajectories belonging to the same route.

**Figure 3:**
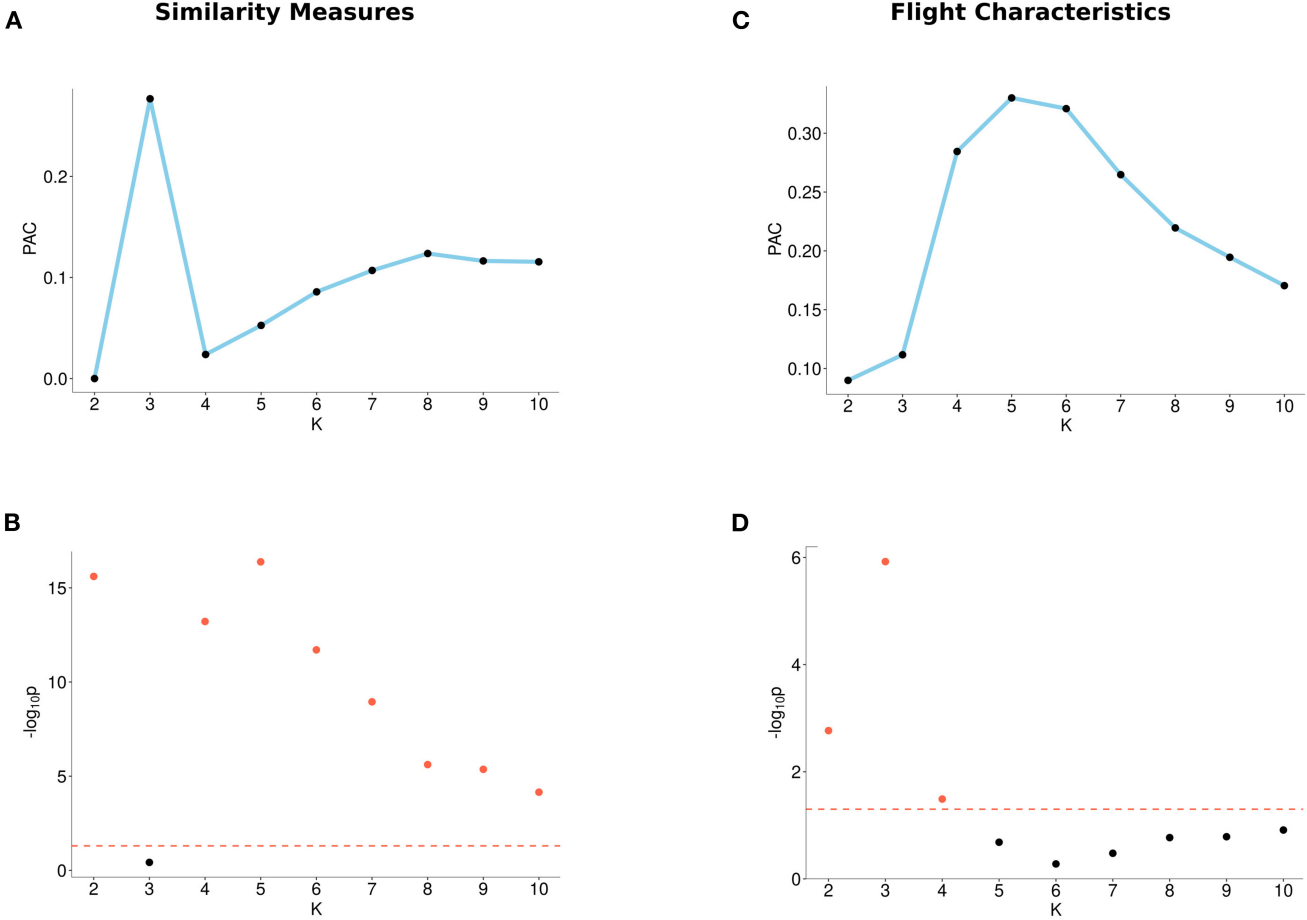
Output from the M3C algorithm based on similarity measures (left) and flight characteristics (right). **(A)** PAC-scores (Proportion of Ambiguous Clustering) of different number of clusters (K) for similarity measures. **(B)** *p*-values of different numbers of clusters (K) for similarity measures. The red dotted line indicates the 0.05 significance level, where points (plotted in red) reach the significance level and points below the line (plotted in black) do not. **(C)** PAC-scores of different numbers of clusters (K) for flight characteristics. **(D)** *p*-values of different numbers of clusters (K) for flight characteristics. The red dotted line indicates the 0.05 significance level, where points (plotted in red) reach the significance level and points below the line (plotted in black) do not.

When using flight characteristics, we found that two clusters (*K* = 2) have a local minimum PAC-score and are significant. When using DTW, Fréchet, or both path similarity measures, we observed local minima of the PAC-score, at *K* = 2, at *K* = 4, and at *K* = 2 and *K* = 4, respectively (for single measure, see Supplementary Figure 2, for both measures, see Figure 3). Furthermore, the *p*-values for these numbers of clusters are below 0.05.

Thus, we found a significant number of clusters of trajectories described by either flight characteristics or path similarities. We have therefore different potential clustering outcomes. To disambiguate between them we will visualize the clusters with t-SNE.

### 3.3. Visualization of Clusters

**Figure 4:**
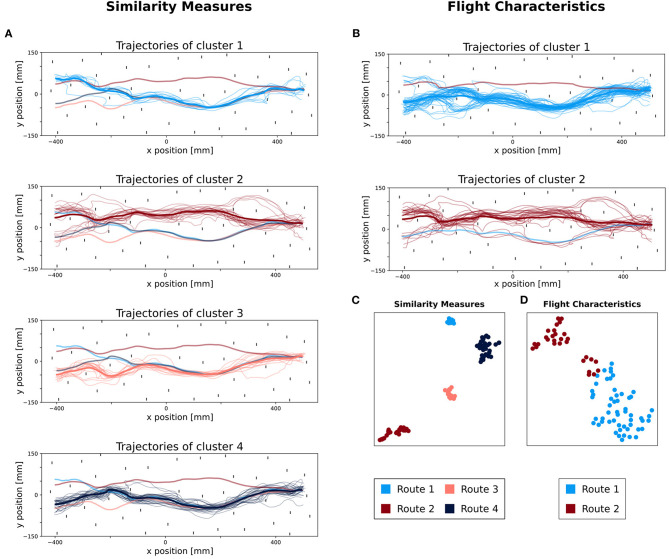
Comparison of classified trajectories between similarity measures and flight characteristics. The four different colors represent the different clusters. **(A)** Top view of all trajectories classified with similarity measures. In each subplot, the trajectories belonging to their respective route are plotted, as well as their average trajectory (in a thicker line). In addition, for each other route, the averaged trajectory is added with a lower opacity. **(B)** Top view of all trajectories classified with flight characteristics. In each subplot, the trajectories belonging to their respective route are plotted, as well as their average trajectory (in a thicker line). In addition, for each other route, the averaged trajectory is added with a lower opacity. **(C,D)** Visualization of M3C clustering with t-SNE (t-distributed stochastic neighboring embedding). **(C)** t-SNE plot of trajectories classified by using similarity measures. **(D)** t-SNE plot of trajectories, classified by using flight characteristics.

We have grouped the trajectories of bumblebees into similar routes by using the M3C method, yielding high dimensional data. To visualize such high dimensional data, they can be projected onto a 2D space by using linear (e.g., Principal Component Analysis) or non-linear projection (e.g., t-distributed stochastic neighboring embedding: t-SNE). Here, we used t-SNE to visualize the two path similarity measures (a 166D space) and the flight characteristics (a 7D space) in a 2D space, respectively. The data points are then labeled according to their corresponding clusters derived from the M3C. The path similarities projected onto a 2D space formed four clusters matching the clusters derived from the M3C (Figure 4C). In contrast, the projection of the flight characteristics in a 2D space does not form such distinct and spatially apart clusters (Figure 4D). One may observe two clusters, but one of them contains points associated to the two routes, hence the clustering outcome is not validated by t-SNE. Visualization with t-SNE indicated *K* = 4 but not *K* = 2 clusters as the M3C for the path similarities and flight characteristics, respectively.

Interestingly, the clustering outcome with *K* = 4 resulted in a split of one of two clusters with *K* = 2. The partitioning of the cluster was thus conserved between *K* = 2 and *K* = 4 (see Supplementary Figure 5).

### 3.4. Visualization of Routes

The last step of our method is to visualize the labeled paths and an average route representing the derived route structure. We plotted each cluster of trajectories, based on flight similarity. We can see that the trajectories assigned to each cluster are spatially closer to one another than to those of the other derived routes (Figure 4A). When visualizing the routes obtained from clustering based on flight characteristics (Figure 4B), the second cluster contains dissimilar paths. Overall, the trajectories grouped based on path similarity form visually coherent groups. The same is not true for the grouping based on flight characteristics.

### 3.5. Effect of Re-sampling Trajectories

We re-sampled our trajectories to reduce the computational demand while preserving the shape of the trajectories by using a constant traveling speed (re-sampling coefficient). Nevertheless, the re-sampling may impact the classification results. To assess the impact of the re-sampling coefficient, we performed clustering for different re-sampling coefficients.

We classified pairs of trajectories for two classification methods (reference *s* = 6 mm/frame and alternative re-sampling *s* ≠ 6 mm/frame). By building a confusion matrix from this classification, we derived the precision of the alternative re-sampling. A precision of one means that pairs of trajectories are sorted in the same manner for both the ground truth and the alternative re-sampling.

**Figure 5:**
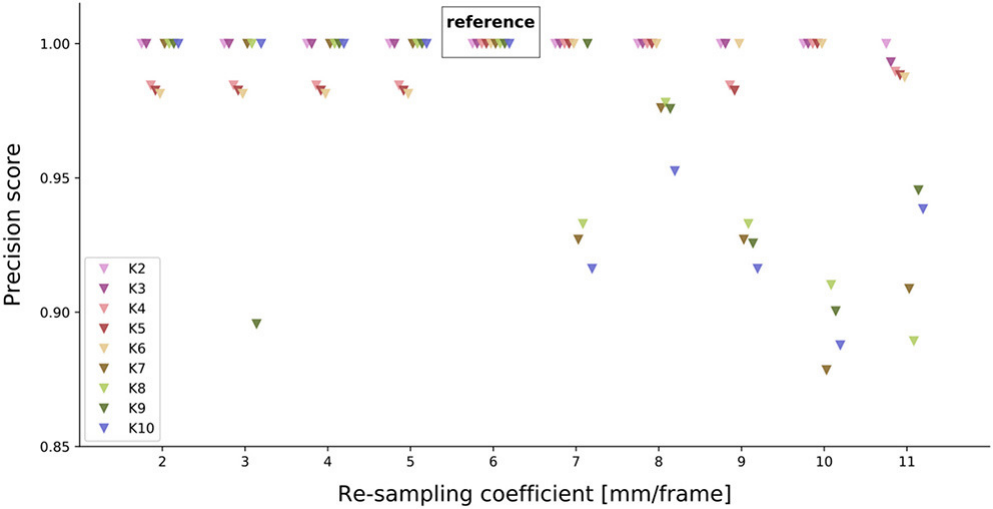
Effect of different re-sampling coefficients on the precision score. For each re-sampling coefficient (x-axis), the precision score for each possible number of clusters was tested. *K* = 6 serves as a reference (truth values) for the other re-sampling coefficients.

We observe that the precision is close to 1 across the tested range of re-sampling parameters (Figure 5), especially for *K* = 4 clusters, the chosen reference number of clusters. However, this precision score may have been obtained by chance. Therefore, we statistically test how likely the precision comes from a random clustering of paths. We observe that the simulated precision scores are distinctly below our tested precision scores (Supplementary Figure 6). Thus, the precision scores obtained from the different re-sampling parameters, are significantly different from a random clustering of trajectories. Since the precision scores are close to 1 and significantly different from random clustering, the re-sampling parameters do not strongly impact the classification results.

## 4. Discussion

We developed a quantitative method to derive routes from groups of trajectories. The number of potential routes was chosen based on the proportion of ambiguous clusters and statistical tests assessing the plausibility of multiple routes among our collection of trajectories. We described trajectories and then clustered them based on their descriptors using Monte Carlo Reference-based Consensus Clustering (John et al., 2020). Path similarity measures (DTW and Fréchet measures) yielded meaningful clusters of trajectories (i.e., routes). In contrast, clustering results based on DTW or Fréchet similarity measures alone were not validated by the t-SNE visualization (Supplementary Figure 2). The same was observed for clustering based on flight characteristics (e.g., average speed, and average lateral position). Concluding only on the result of M3C may lead to ambiguous results, as a low PAC-score and a rejection of the null hypothesis (i.e., having only one cluster) may be found for different numbers of clusters. By using M3C on two path similarity measures and visualizing the results with t-SNE, we could determine a potential number of routes in the trajectories of bees.

Computing the path similarity between a pair of trajectories is time consuming. The complexity of the algorithm often grows as a product of the length of the two trajectories (*L*_1_ and *L*_2_). Since we calculated the similarities between all pairs of *N*_*t*_ trajectories of average length $\tilde{L}$, the complexity was in the order of ${\left(\tilde{L}\times N_{t}\right)}^{2}$. We reduced the computational demand by re-sampling every trajectory to lower the number of observations. The re-sampling parameter in our tested range did not strongly affect the resulting classifications.

### 4.1. Alternative Uses of our Approach

The bees in the cluttered environments flew from one end of the tunnel to the other. Our method already takes the distance between the first observations between two paths into account when computing similarity measures (due to a property of DTW). However, in nature, animals will travel between two locations in both directions. The route followed by the animal may differ between an inbound and outbound journey (as was observed in ants, Kohler and Wehner, 2005). Comparing an inbound path with an outbound path without mirroring, will lead to different routes, even if the paths visually overlay in space. Clustering the animal's inbound and outbound journey requires to mirror either the inbound or outbound paths so that they start at the same location.

In addition, in nature animals may slightly deviate from their route, for example by being pushed by a gust of wind (Riley et al., 1999; Wystrach and Schwarz, 2013; Ravi et al., 2016). The larger the deviations are, the smaller the similarities between paths become. Thus it may lead to classifications of such trajectories into different routes. Using partial match measures such as the LCSS distance (see Su et al., 2020 for review) lower the risk of classifying several disturbed trajectories belonging to the same route into different routes.

### 4.2. Associating Novel Trajectories to Clusters

Understanding the underlying mechanisms driving animals through their environment often involves building a model of the perception-behavior loop and simulating an agent moving in the environment. However, when the originally observed trajectories are inherently variable (e.g., Lobecke et al., 2018), it becomes difficult to assess whether an artificial agent mimics, at least to some extent, the animal's behavior. Furthermore, simulated trajectories might differ between runs (for example due to intrinsic noise in the model, e.g., Bertrand et al., 2015; Le Möel and Wystrach, 2020), which might differ to some extent from the animal's behavior. For a route-following agent, one would be satisfied, if the same number of routes can be derived from the agent's trajectories, as were derived from the animal's trajectories. Our clustering method can be used to address these aspects. First, as we did here, routes can be extracted from a collection of experimentally observed trajectories. Second, the same procedure can be applied on modeled trajectories to assess whether the descriptions of these simulated trajectories also cluster into the same routes as the experimentally determined trajectories. Third, we can map the trajectories of the modeled agent to the cluster of the animal's trajectories (or vice versa). Indeed, our method relies on a classifier (e.g., partition around medoids) using trajectory similarities. By calculating the similarity between an agent's trajectory (or any novel trajectory) and those of an animal, the agent's trajectory becomes a point in the input space of the classifier. Thus, we can assign it to one of the clusters, i.e., one of the routes of the animals. Therefore, we can compare an agent path with the behavior by using our method.

### 4.3. Clustering Trajectories of Non-route Following Behaviors

We developed our method to derive routes from trajectories. However, we can extend it to trajectories that do not form routes. For example, animals may steer in a given direction to go away from a food source and hide its collected reward (e.g., dung beetle, Dacke et al., 2013), move in a convoluted manners to avoid a predator or chase prey (Boeddeker et al., 2003; Kane and Zamani, 2014; Wardill et al., 2017) or perform complex search behavior when searching for home (Doussot et al., 2020; Schultheiss et al., 2015). In these examples, the animals are not following a route. However, one may be interested in the similarities between trajectories. One may seek to statistically group the runs of multiple dung beetles or the convoluted paths of chasing flies. The series of turns may be more important than the actual position of the animal when comparing such trajectories. Instead of using position based similarity measures (such as DTW and Fréchet), one may use similarity measures based on the direction of movements of the animals (e.g., SPADE, Chen et al., 2007).

Taken together, by combining trajectory similarities and a clustering approach without knowledge of the number of clusters, common path structures between the trajectories of walking, flying or swimming animals can be identified. We illustrated our method by using flights of bumblebees in cluttered terrain and could extract four common routes. Trajectory classification has applications in several fields (Wang et al., 2020) and is an opportunity to identify common strategies in animal behavior, from maintaining a given direction to following routes, or chasing prey.

## Data Availability Statement

The original contributions presented in the study are included in the data publication (Gonsek et al., 2020): https://pub.uni-bielefeld.de/record/2945187.

## Author Contributions

AG and OB conceived and designed the method. MJ, SR, and OB designed the bumblebee experiments. MJ and SR conducted and supervised behavioral experiments. AG implemented the method and analyses. MJ and SR reviewed the implemented method. AG, MJ, and OB curated the behavioral experiments. All authors contributed to writing and revising the manuscript.

## Conflict of Interest

The authors declare that the research was conducted in the absence of any commercial or financial relationships that could be construed as a potential conflict of interest.
