# Supplementary material for: From Paths to Routes: A Method for Path Classification
Source: Front Behav Neurosci. 2021 Jan 21;14:610560. doi: 10.3389/fnbeh.2020.610560 (PMC7859641; doi:10.3389/fnbeh.2020.610560)
Supplement: Supplementary file 1 [file Data_Sheet_1.pdf]

Supplementary Material

1 SUPPLEMENTARY TABLES AND FIGURES

1.1 Figures

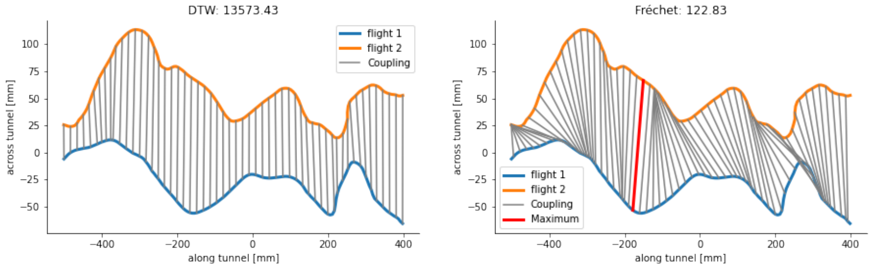

Figure S1. Example of DTW and Fréchet between two flights of bumblebees. The DTW results from the sum of the drawn connections (in gray) between the two trajectories. Fréchet distance is the longest connection between the two trajectories. The connections differ between the two methods, because whereas DTW minimises the total length of connection, Fréchet finds the minimum longest connection to connect the two trajectory.

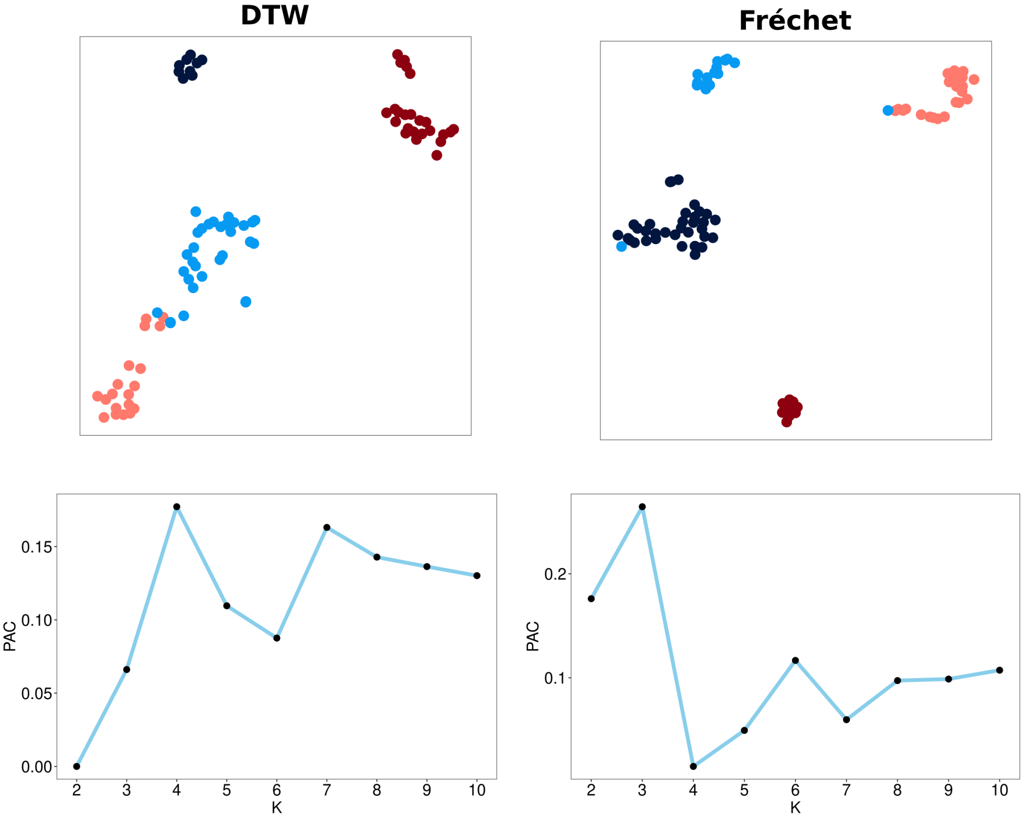

Figure S2. T-SNE visualisation and PAC-scores of the M3C clustering results with only the DTW (left) or the Fréchet measure (right), respectively.

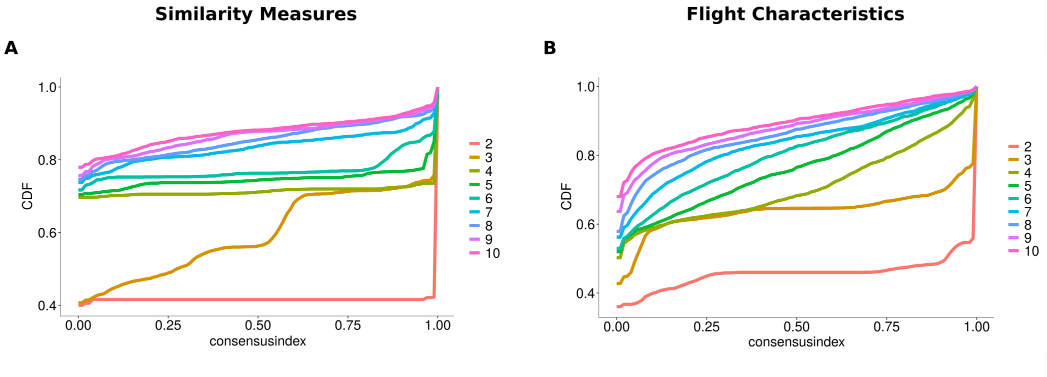

Figure S3. Scores of the cumulative distribution functions (CDF) of the M3C results for the similarity measures (**A**) and the flight characteristics (**B**). Each line represents the CDF-plot for a certain number of clusters in between the range of $K \in [2, 11]$.

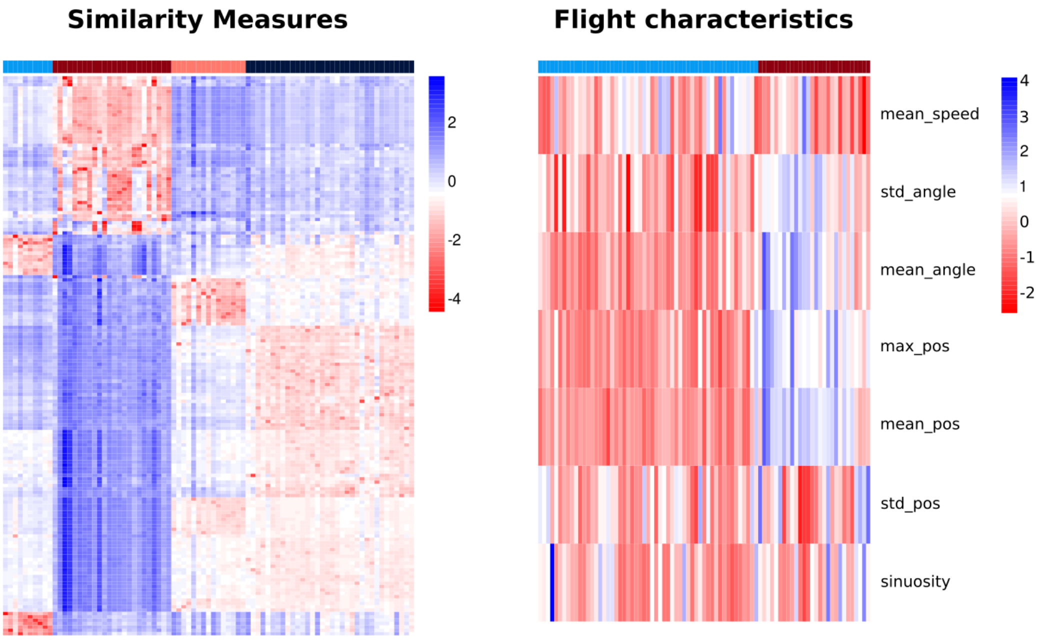

Figure S4. (left) sorted heatmap of normalised distance values of both similarity measures (DTW and Fréchet), where the columns represent the trajectories ,and the rows the respective paired trajectories for both measures. (right) sorted heatmap of normalised flight characteristic values. The columns represent the trajectories and the rows depict the flight characteristic values.

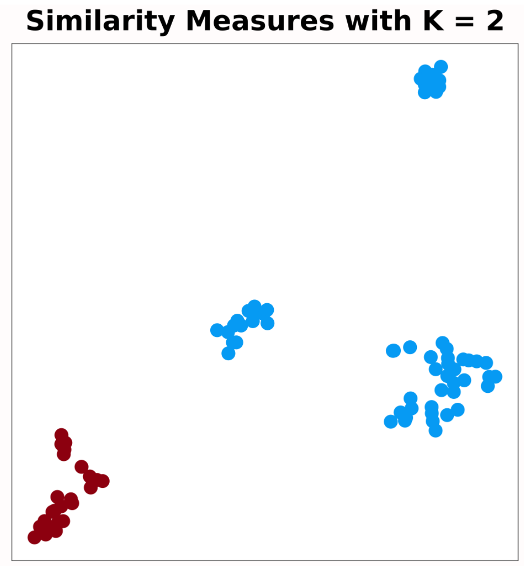

Figure S5. T-SNE visualisation of the M3C clustering with the DTW and the Fréchet measure, here with K = 2. We observe that one the blue cluster is splitted into three clusters when using K=4

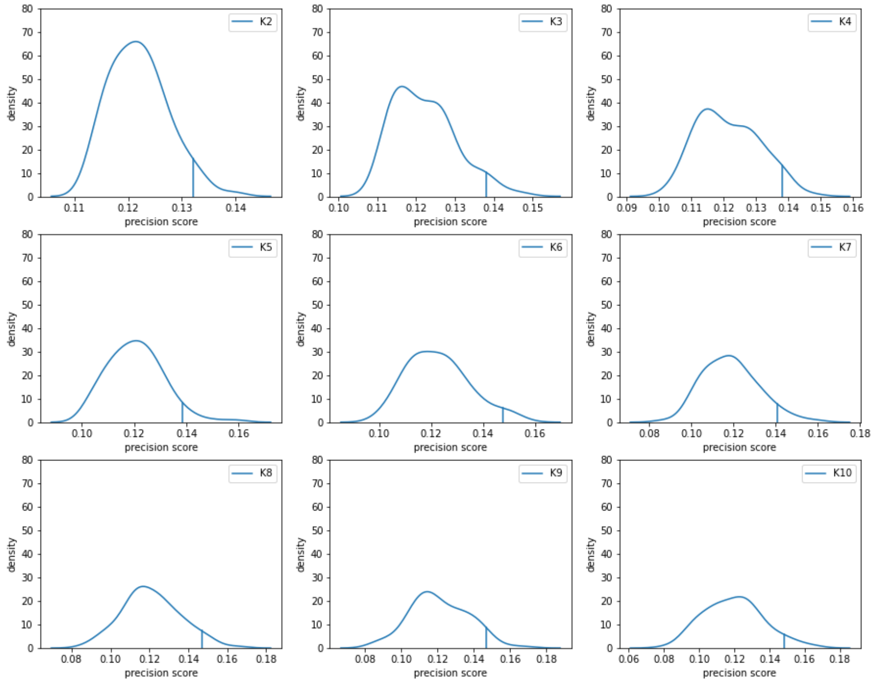

Figure S6. Distribution of precision scores of repeated runs between our clustering with $s = 6$ and 100 random clustering. Our method investigated $K = 2$ to $K = 10$ potential clusters. Each subplot is for a given number of cluster $K \in [2, 10]$. The vertical line represents the 95th percentile of the distribution.
